# Supplementary material for: Fine Mapping of Stripe-Rust-Resistance Gene YrJ22 in Common Wheat by BSR-Seq and MutMap-Based Sequencing
Source: Plants (Basel). 2022 Nov 25;11(23):3244. doi: 10.3390/plants11233244 (PMC9740260; doi:10.3390/plants11233244)
Supplement: Supplementary file 1 [file plants-11-03244-s001.zip › plants-1934693-supplementary.pdf]

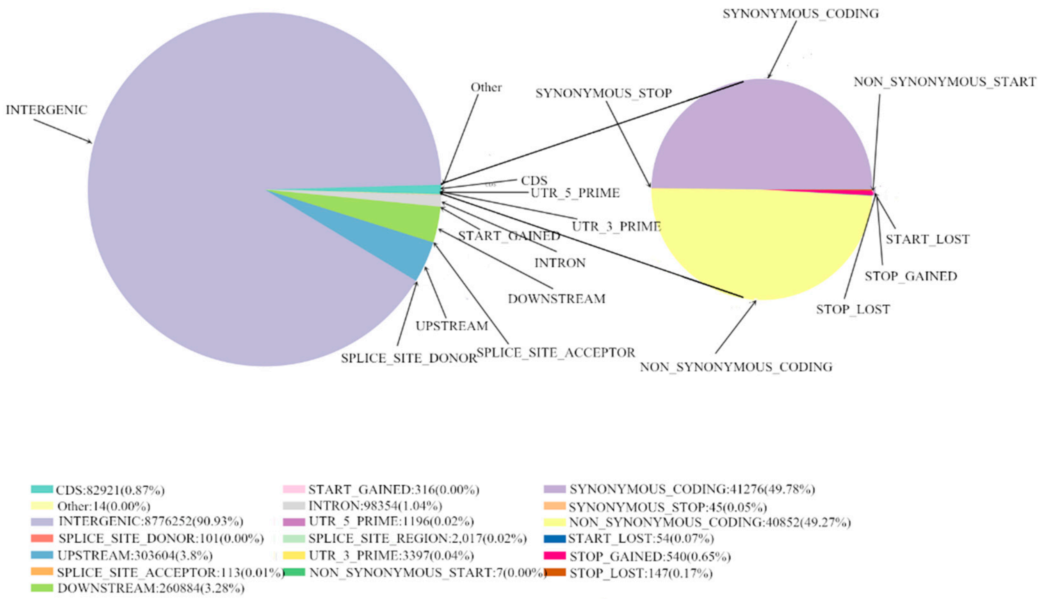

Figure S1 Statistical results of candidate SNP annotations

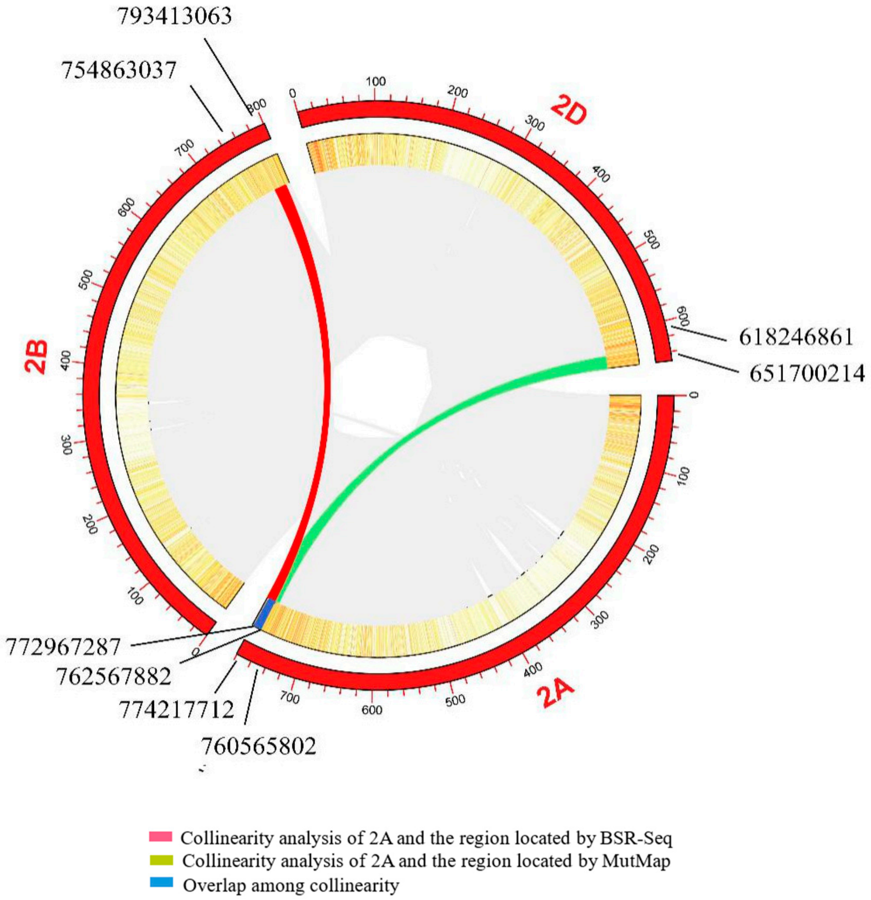

Figure S2 Collinearity analysis of wheat chromosomes 2A, 2B, and 2D

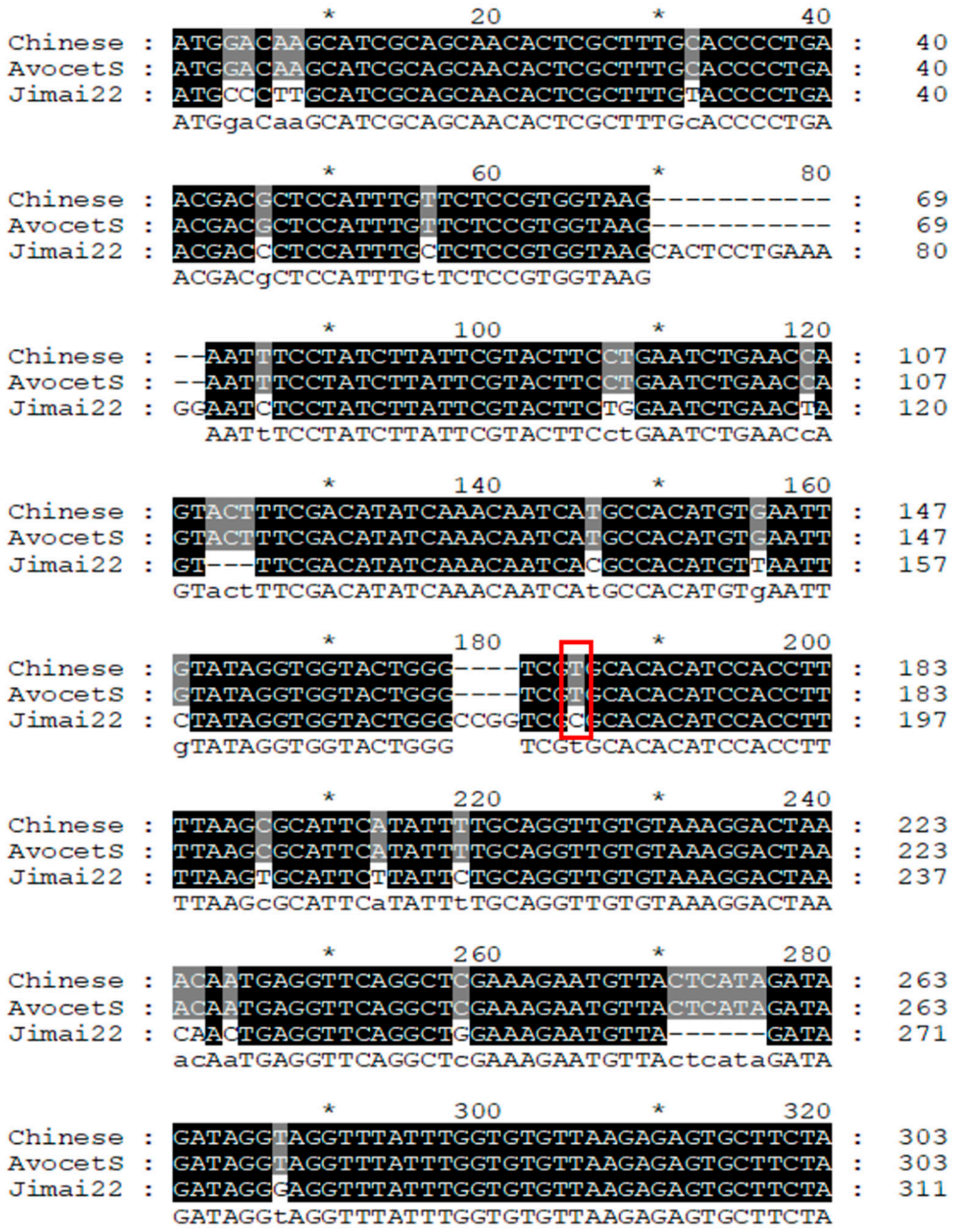

(a)

```
                        *        20         *        40
Jimai22 : MPLHRSNTRFVPLNDPPFALRGASNSSFNNMDNLRRSSGIGQ :  42
AvocetS : MDKHRSNTRFAPLNDAPFVLRGASNSSFNNMDNLRRSSGIGQ :  42
          M  HRSNTRF PLND PF LRGASNSSFNNMDNLRRSSGIGQ

                      *        60         *        80
Jimai22 : TKSHASSPLGALRQKMPPSGNRSLHTSRPLSAPVANRPLSPH :  84
AvocetS : TKSHASSPLGALRQKMPPSGNRSLHTSRPLSAPVANRPLSPH :  84
          TKSHASSPLGALRQKMPPSGNRSLHTSRPLSAPVANRPLSPH

                    *       100         *       120
Jimai22 : LPLKKPQLSSTFSISHRIFGVALGAAIISIPLATKFSLMFGV : 126
AvocetS : LPLKKPQLSSTFSISHRIFGVALGAAIISIPLATKFSLMFGV : 126
          LPLKKPQLSSTFSISHRIFGVALGAAIISIPLATKFSLMFGV
```

(b)

Figure S3 (a) Sequence alignment of *TraesCS2A02G573200* between Jimai 22 and Avocet S, (b) Alignment of the deduced amino acid sequences of TraesCS2A02G573200 between Jimai 22 and Avocet S
